# Context-dependent differences in the functional responses of Lactobacillaceae strains to fermentable sugars

**DOI:** 10.3389/fmicb.2022.949932

**Published:** 2022-10-24

**Authors:** Ronit Suissa, Rela Oved, Harsh Maan, Uzi Hadad, Omri Gilhar, Michael M. Meijler, Omry Koren, Ilana Kolodkin-Gal

**Affiliations:** ^1^Department of Molecular Genetics, Weizmann Institute of Science, Rehovot, Israel; ^2^Department of Chemistry, Ben-Gurion University of the Negev, Be’er Sheva, Israel; ^3^Ilse Katz Institute for Nanoscale Science and Technology, Ben-Gurion University of the Negev, Be’er Sheva, Israel; ^4^Azrieli Faculty of Medicine, Bar-Ilan University, Safed, Israel; ^5^Department of Plant Pathology and Microbiology, Faculty of Agriculture, Food and Environment, The Hebrew University of Jerusalem, Rehovot, Israel

**Keywords:** aggregation, acid stress, glucose, probiotics, lactobacillacea, flow cytometry, biofilms

## Abstract

Lactobacillaceae are Gram-positive rods, facultative anaerobes, and belong to the lactic acid bacteria (LAB) that frequently serve as probiotics. We systematically compared five LAB strains for the effects of different carbohydrates on their free-living and biofilm lifestyles. We found that fermentable sugars triggered an altered carrying capacity with strain specificity during planktonic growth. In addition, heterogeneous response to fermentable sugar was manifested in microbial aggregation (measured by imaging flow cytometry), colony development, and attachment to mucin. The acid production capacities of the strains were compatible and could not account for heterogeneity in their differential carrying capacity in liquid and on a solid medium. Among tested LAB strains, *L. paracasei, and L. rhamnosus GG* survived self-imposed acid stress while *L. acidophilus* was extremely sensitive to its own glucose utilization acidic products. The addition of a buffering system during growth on a solid medium significantly improved the survival of most tested probiotic strains during fermentation, but the formation of biofilms and aggregation capacity were responsive to the carbohydrate provided rather than to the acidity. We suggest that the optimal performance of the beneficial microbiota members belonging to Lactobacillaceae varies as a function of the growth model and the dependency on a buffering system.

## Introduction

Firmicutes are a dominant phylum in the human microbiota, and the proportions of members of this phylum vary between individuals and are greatly influenced by the local pH (Spor et al., 2011). Accordingly, the stomach is the least diverse growth niche within the human gastrointestinal tract (due to its extreme acidity). In the microbiome, the presence of probiotic strains varies between healthy individuals. Probiotics are defined simply as “microorganisms that, when administered in adequate amounts, confer a health benefit to the host” (Qin et al., 2010). Probiotic strains are consumed either as fresh fermentation products or as dried bacterial supplements, with Lactobacillaceae and Bifidobacteria, being the two most widely used probiotic species (Didari et al., 2014; Azad et al., 2018). The main strains currently used for probiotic formulation were originally isolated from fermented products or humans (Fontana et al., 2004; Vijaya Kumar et al., 2015; Zielińska et al., 2018).

Lactobacillaceae are Gram-positive rods, facultative anaerobes, and belong to the lactic acid bacteria (LAB) group, as lactic acid is their main end-product of carbohydrate metabolism (Bintsis, 2018; Wang et al., 2021). This family is naturally found in the gastrointestinal tract (GIT) of humans and animals as well as in the urogenital tract of females (Turroni et al., 2014). LAB are considered efficient fermenters, proficient in the production of energy under anaerobic conditions, or when oxygen is limited. The fermentation process involves the oxidation of carbohydrates to generate a range of products including organic acids, alcohol, and carbon dioxide (Hofvendahl and Hahn-Hägerdal, 2000). In general, the response of all LAB strains to fermentation is considered to be uniform and primarily depends on the capacity to utilize glucose and its products (Qin et al., 2010).

In LAB, a phosphotransferase system (PTS) transports glucose across the membrane that is then used by the glycolysis pathway to produce pyruvate. This pathway generates energy and consumes NAD^+^. Pyruvate is then converted into L-and D-lactate by the stereospecific NAD-dependent lactate dehydrogenases (LDHs), LdhL and LdhD, respectively, which regenerate NAD^+^ and maintain the redox balance (Ferain et al., 1994). In the presence of oxygen and following the depletion of glucose, lactate is oxidized to pyruvate *via* the enzyme lactate oxidase followed by the production of acetate using pyruvate oxidase and acetate kinase (ACK) enzymes. The formation of acetate as the major fermentation end product results in homoacetic fermentation (Hols, 2004). Depending on the utilized saccharide and the environmental conditions, carbohydrate catabolism can differ between the LAB species and specific strains. LGG can catabolize glucose and mannose but not raffinose and xylose (Hedberg et al., 2008). *Lacticaseibacillus casei* ATCC 393 and *Lactiplantibacillus plantarum* ATCC 8014 can utilize glucose (Paucean et al., 2013), while *Lactobacillus acidophilus* ATCC 4356 and *L. casei* ATCC 393 can catabolize both glucose and raffinose from soymilk (Yeo and Liong, 2010). In contrast, *Lacticaseibacillus paracasei* fermentation capacity was higher with glucose compared with raffinose (Hedberg et al., 2008; Palacio et al., 2014).

In addition to the importance of glucose utilization to microbial energy production, different regulatory effects for fermentable and non-fermentable carbohydrates were episodically reported. Glucose and fructose induce biofilm formation in *Lacticaseibacillus rhamnosus GG,* together with changes in protein abundance and surface proteome (Savijoki et al., 2019). *L. plantarum* increases biofilm formation when supplemented with manganese and glucose (Salas-Jara et al., 2016) and *Lactobacillus acidophilus* NCFM improves its adhesive properties upon raffinose utilization together with changing its proteome architecture (Celebioglu et al., 2016). Altogether, these findings indicate that changes in carbon sources and their concentrations induce more complex adaptations than alterations in microbial growth and that these adaptations need to be systematically explored, characterized, and compared to predict accurately the optimal compositions and formulations of probiotics.

To map adaptations to carbohydrates that are growth-dependent and independent, we systematically compared the response to metabolic stress in microaerophilic (CO_2_ > 3%) conditions of the five probiotic Lactobacillaceae species: *Lacticaseibacillus rhamnosus GG* (Segers and Lebeer, 2014)*, Lacticaseibacillus casei* (Karapetsas et al., 2010)*, Lactobacillus acidophilus* (Huang et al., 2022)*, Lacticaseibacillus paracasei,* and *Lactiplantibacillus plantarum* (Rocchetti et al., 2021) during planktonic growth and colony biofilm formation. *Bacillus coagulans*, a probiotic *Bacilli* belonging to the same phylum (Cao et al., 2020), was studied as a non-Lactobacillaceae LAB control strain. Under our conditions, similar glucose utilization efficiencies were observed between the species (as judged by the levels of the end products: lactate and acetate). Our results indicate that a differential response to carbohydrates is correlated with a differential acid tolerance, rather than a differential production of organic acids. Thereof, LAB bacteria significantly differ in their response to their own self-imposed acid stress from glucose utilization products and can be clustered into fermentation resistant and fermentation sensitive strains. The differential adaptation to acidic products included reversible changes in cellular organization and colony formation.

## Results

### Fermentable sugars specifically but heterogeneously affect the carrying capacity during planktonic growth

**Figure 1:**
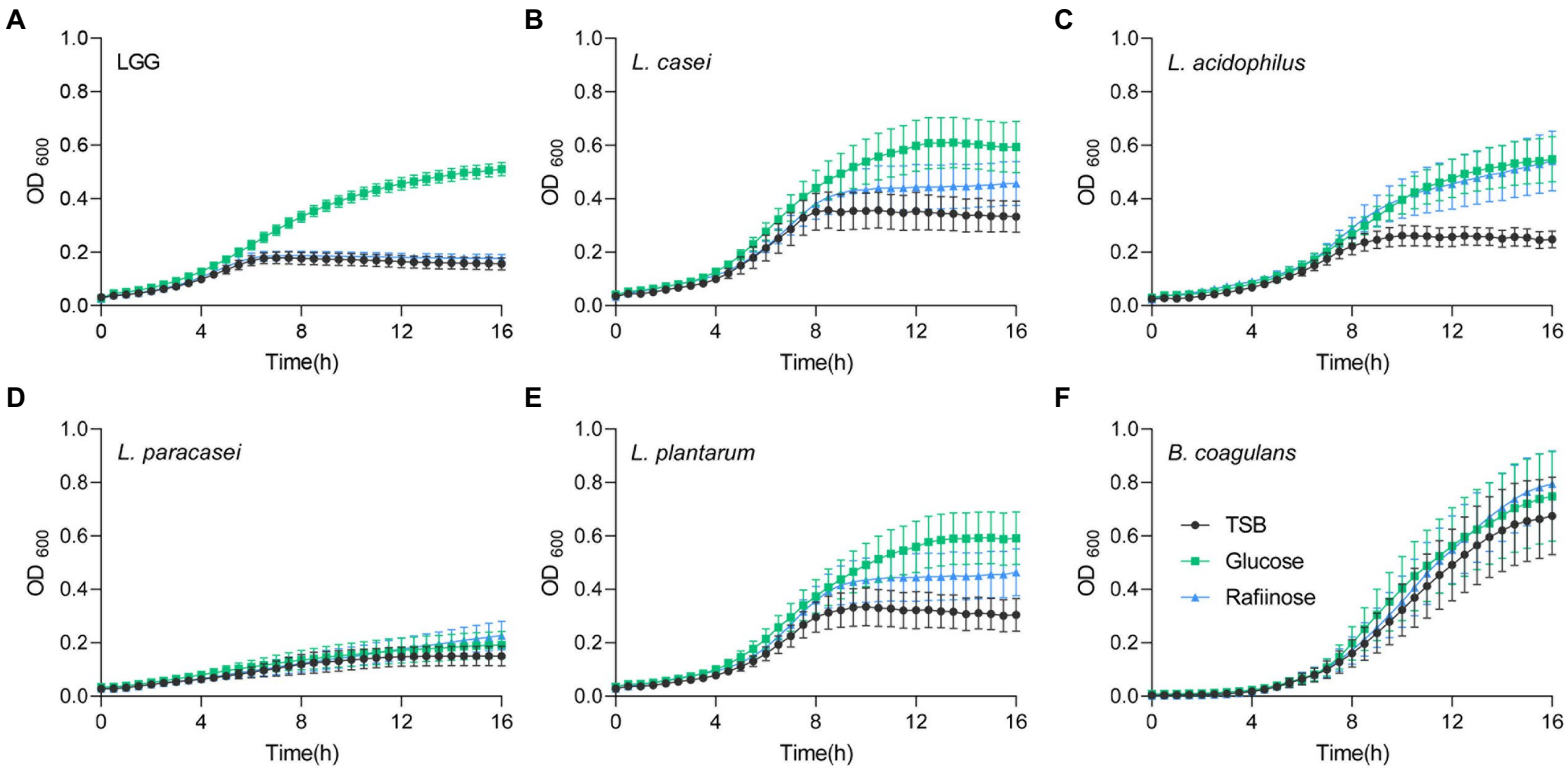
Heterogeneous alterations in planktonic growth of LAB strains during glucose utilization. Planktonic growth of the indicated species **(A)** LGG; **(B)** *L. casei*; **(C)** *L. acidophilus*; **(D)** *L. paracasei*; **(E)** *L. plantarum*, and **(F)** *B. coagulans* in TSB medium (control) either supplemented or not with glucose (1% W/V) and raffinose (1% W/V). Graphs represent mean ± SEM from six independent experiments (*n* = 24).

**Figure 2:**
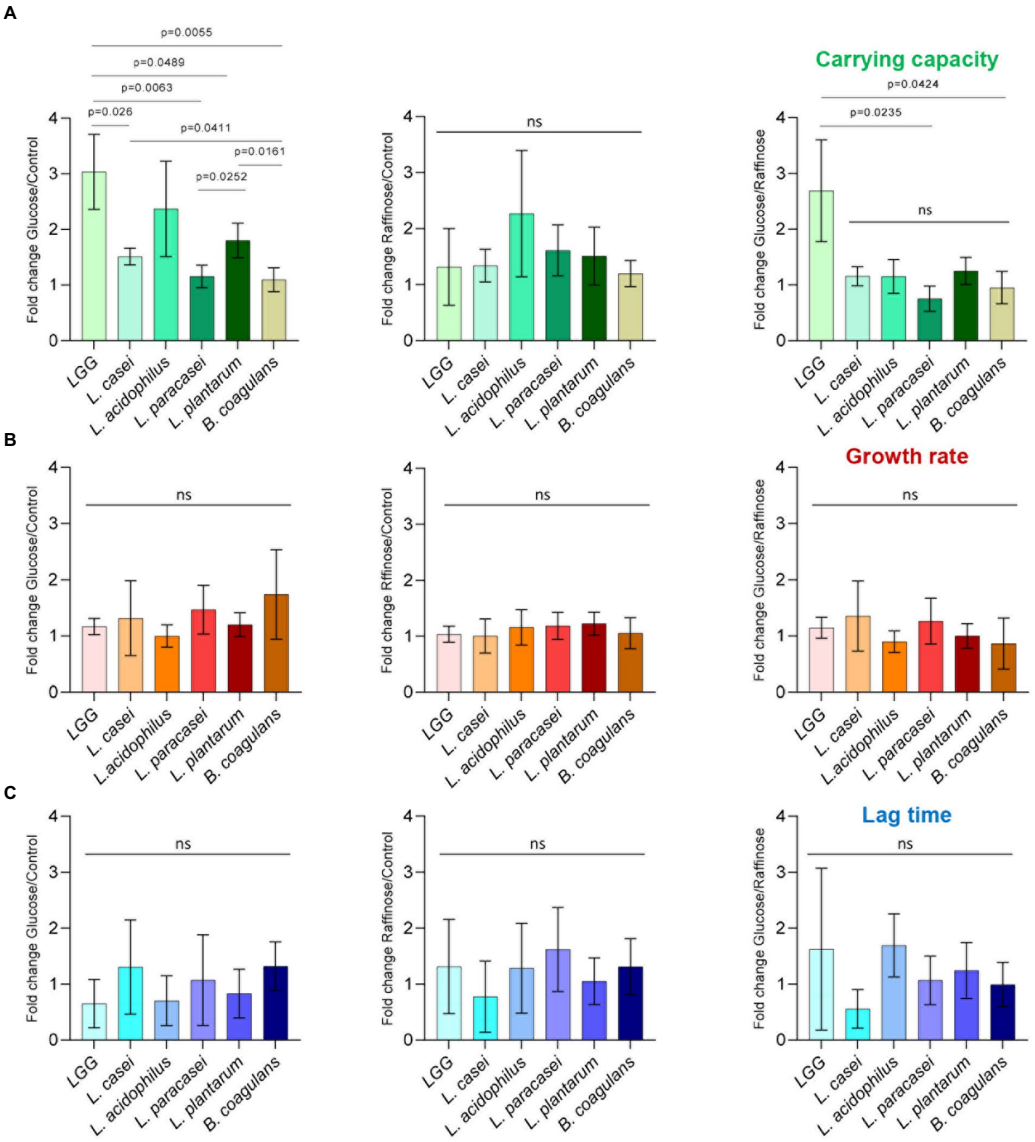
Carbon-dependent growth induction is specifically manifested by an altered carrying capacity. Analysis of planktonic growth from the data shown in Figure 1 using GrowthRates 3.0. **(A)** Fold change, in carrying capacity, **(B)** growth rate, and **(C)** lag time of indicated species between glucose/control, raffinose/control, and glucose/raffinose. Graphs represent mean ± SD from six independent experiments (*n* = 24). Statistical analysis was performed using Brown-Forsythe and Welch’s ANOVA with Dunnett’s T3 multiple comparisons test. *p* < 0.05 was considered statistically significant.

To test whether fermentable sugars affect growth differently than non-fermentable sugars, five probiotic Lactobacillaceae species were grown on a rich medium (TSB) in a shaking culture either with glucose (fermentable) or with raffinose (non-fermentable). All bacteria grew similarly in the rich medium. The addition of glucose induced growth as judged by an increased carrying capacity (reflected by the maximal OD measured) of LGG and *L. acidophilus* compared to TSB alone, but its effect on growth in *L. casei*, *L. paracasei*, *L. plantarum*, and *B. coagulans* was extremely noisy (Figure 1). To better distinguish between the growth patterns of the different probiotic strains, we further analyzed growth with the software GrowthRates 3.0. We extracted the length of the lag phase, the growth rate, and the carrying capacity of all LAB strains with the different treatments (Hall et al., 2014). Our results indicated that the heterogeneous response was primarily manifested in an altered carrying capacity (e.g., the maximal OD of the cultures; Figure 2A). Strains showed varied carrying capacities from each other, and indeed LGG and *L. acidophilus* had enhanced carrying capacity in response to Glucose, while other probiotic strains did not exhibit a significant response. In all species, the growth rate and lag time were not significantly altered by glucose addition (Figures 2B,C).

We then asked whether the induction of growth is an outcome of fermentable sugar utilization, as fermentation is a metabolic process allowing the production of energy under anaerobic conditions. To answer this, we assessed the growth of the bacteria on media supplemented with raffinose, a sugar source less compatible with fermentation, as it was indicated that alpha-galactosidase, responsible for the hydrolysis of this sugar, is not enzymatically active (Garro et al., 1998; Hedberg et al., 2008; Zartl et al., 2018). Raffinose did not induce the growth of LGG, suggesting that the induction of growth depends on the fermentable nature of the sugar (Figures 1, 2A). In contrast, the carrying capacity of *L. acidophilus* cultures increased with both glucose and raffinose (Figure 1) but failed to meet the statistical significance of each parameter of growth (Figures 2B,C). In general, the addition of raffinose failed to induce significant alterations in growth parameters in all strains. Similar results were observed when the bacteria were grown with mannose (fermentable) which induced enhanced carrying capacity and xylose non-fermentable), which failed to induce significant alterations of growth (Supplementary Figure S1). With the exception of carrying capacity, other parameters of growth remained unaltered by sugar application in all strains (Figures 1, 2; Supplementary Figure S1).

### Differential response of Lactobacillaceae to fermentation is not a result of organic acid production

**Figure 3:**
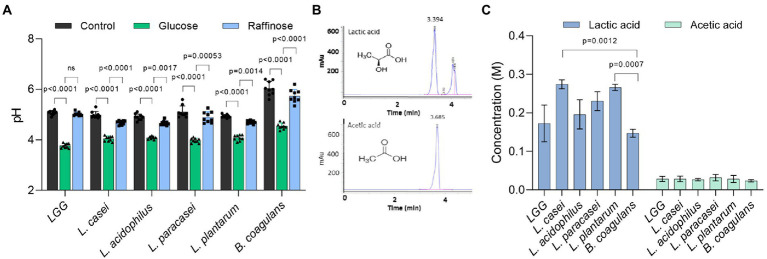
The variation at the species level in growth enhancement is not due to glucose catabolism. **(A)** The pH of the conditioned media of indicated species in TSB medium (control) and TSB medium supplemented with glucose (1% W/V) and raffinose (1% W/V). Statistical analysis was performed using two-way ANOVA followed by Dunnett’s multiple comparison test. *p* < 0.05 was considered statistically significant. **(B)** HPLC chromatograms indicate an exclusive peak and stable retention time for the pure standards of lactic acid and acetic acid. **(C)** The concertation of lactic acid and acetic acid produced by the indicated species. Bacteria were grown on an MSgg medium supplemented with glucose (1% W/V) for 24 h. The conditioned medium from the cultures was collected and analyzed using the C-18 column. Statistical analysis was performed using Brown-Forsythe and Welch’s ANOVA with Dunnett’s T3 multiple comparisons test. *p* < 0.05 was considered statistically significant.

To compare the fermentation efficiency, we measured the acidity of the medium after growth in the presence and absence of glucose. Indeed, after 24 h the pH of the medium decreased in both glucose and raffinose compared to TSB alone (Figure 3A). The acidity of Lactobacillaceae conditioned medium grown in the absence of glucose and the addition of raffinose was approximately 4.5–5, while the addition of glucose to the medium lowered the pH to 4 or less confirming that upon application of glucose, the acidification of the growth media was enhanced in all Lactobacillaceae that were tested. Compared with other tested strains, *B. coagulans* altered the pH the least, e.g., to 4.5 in the presence of glucose and 6 in its absence (Figure 3A). The pH drop in the growth medium source was comparable between all Lactobacillaceae strains in glucose and raffinose, except in LGG which was inert to raffinose.

To confirm that the uniform drop in pH levels resulted from the differential accumulation of organic acids among the tested LAB species, we measured the accumulation of glucose utilization products (organic acids), focusing on the levels of key organic acids, lactate, and acetate using high-performance liquid chromatography (HPLC; Supplementary Figure S2). In general, lactic acid was ten times more abundant than acetic acid in the growth media. When we statistically analyzed the concentration of lactate produced by the bacteria, there was no single species that was significantly different from the other group members, indicating that the production of organic acids and thereby glucose utilization is performed similarly under our conditions (Figures 3B,C) and cannot account for the differential carrying capacity.

To exclude that the differential response to fermentation is a result of different enzymatic activities of enzymes involved in the process, we studied *in silico* the presence of lactate dehydrogenase from different LAB strains. Alignment of LDH proteins from different Lactobacillaceae members (Supplementary Figure S3) indicated that LGG *L. paracasei* and *L. casei* have two groups of homologues LDH proteins with more than 90% identity. 16S rRNA gene sequence alignment showed that all species have at least 89% similarity, and the evolutionary closest species are LGG *L. casei* and *L. paracasei* based on 16S rRNA gene sequences. Therefore, the key differences revealed by growth analysis on fermentable sugars are despite the high evolutionary closeness of all five species.

### Self-imposed acid stress in biofilm colonies of LAB species

**Figure 4:**
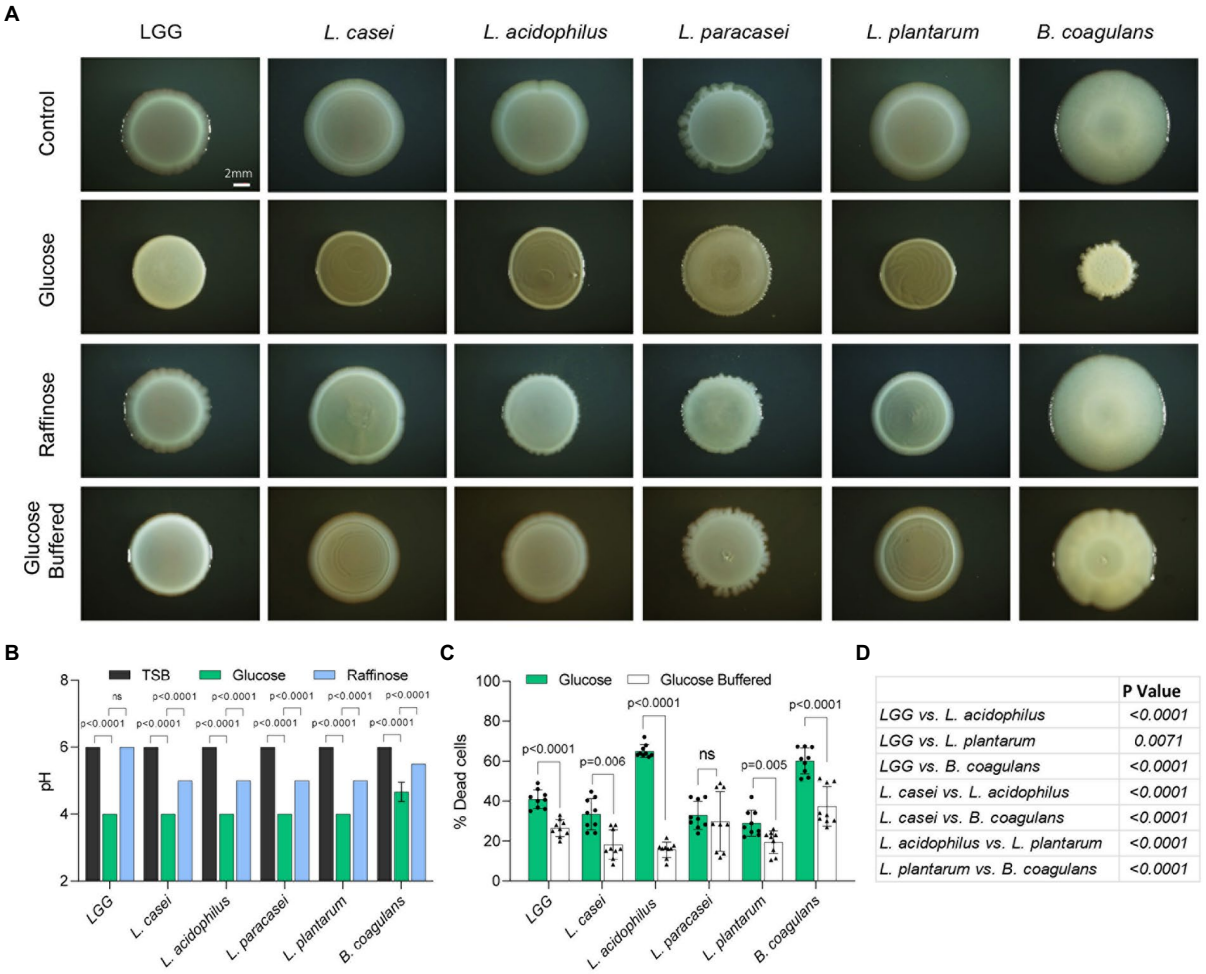
Self-imposed acid stress has a role in the formation of structured microbial colonies. **(A)** Shown are the indicated biofilm colonies grown on TSB medium (control), TSB medium supplemented with glucose and raffinose (1% W/V), and TSB medium supplemented with glucose (1% W/V) + buffer. Biofilms were grown at 37°C in a CO_2_ enriched environment. Biofilm colonies were imaged at 72 h post inoculation. Scale bar = 2 mm. **(B)** Measurement of pH of the indicated species shown in A. Statistical analysis was performed using two-way ANOVA followed by Dunnett’s multiple comparison test. *p* < 0.05 was considered statistically significant. **(C)** Flow cytometry analysis of the number of dead cells of the indicated species shown in **(A)**. Colonies were grown on TSB medium supplemented with glucose (1% W/V), and TSB medium supplemented with glucose (1% W/V) + buffer. Data were collected 72 h post inoculation; 100,000 cells were counted. Y-axis represents the % of dead cells, graphs represent mean ± SD from 3 independent experiments (*n* = 9). Statistical analysis was performed between glucose and glucose + buffer using unpaired two tailed t test with Welch’s correction. *p* < 0.05 was considered statistically significant. **(D)** Table showing multiple comparison tests between the dead cell populations of the indicated species grown in TSB medium supplemented with glucose (1% W/V). Statistical analysis was performed using Brown-Forsythe and Welch’s ANOVA with Dunnett’s T3 multiple comparisons test. *p* < 0.05 was considered statistically significant.

To further study the differential response to fermentation in Lactobacillaceae, we examined the growth on a solid medium under static conditions [frequently correlated with biofilm formation (Povolotsky et al., 2021)], we assessed the colony morphology with the addition of glucose and raffinose (Figure 4A). In all strains, the application of glucose induced the formation of asymmetric, smaller, and morphologically different colonies. Raffinose did not induce the same morphological changes in colony structure, suggesting glucose metabolism has a role also in shaping the colony architecture (Figure 4A). Solid growth media from bacteria grown on TSB or TSB with raffinose had similar pH values of approximately 5.5 (Figure 4B; Supplementary Table S1). The addition of glucose to the growth media lowered the pH significantly suggesting that glucose induced fermentation in all tested strains under these conditions. To test whether acid stress is related to the alterations in colony morphology we monitored cell death using flow cytometry in the presence and absence of a buffering system. As shown, in all strains, with the exception of *L. paracasei*, the buffering system significantly enhanced the survival of the cells, and acid-dependent cell death during fermentation significantly varied between the strains (Figures 4C,D). The addition of a buffer also resulted in colony morphology comparable to the morphology of strains grown without glucose. While cell death was reduced, the buffer did not fully restore the viability of the cells within a colony but was sufficient to restore colony morphologies to those observed in a glucose-free solid medium (Figure 4A). These results may suggest that cell death on solid biofilm media primarily but not solely results from differential acid sensitivity, and that the alterations in biofilm formation capacities occur independently of cell counts.

### Glucose acts as a broad-spectrum regulator of aggregation and adhesion properties while raffinose specifically affects *Lactiplantibacillus plantarum* and *Lactiplantibacillus acidophilus*

**Figure 5:**
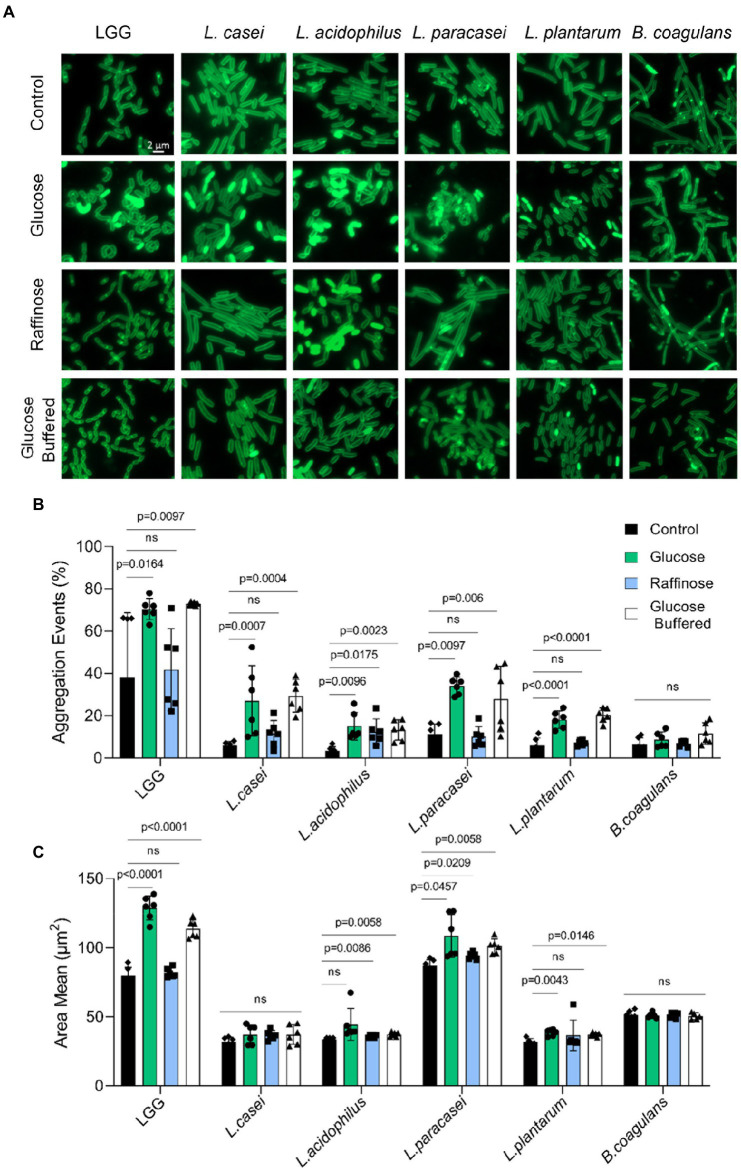
Carbohydrates specifically regulate microbial aggregation. **(A)** Fluorescence microscope images of cells from biofilm colonies that were grown on TSB medium (control), TSB medium supplemented with glucose and raffinose (1% W/V), and TSB medium supplemented with glucose (1% W/V) + buffer. Cells were stained using Green-membrane stain FM™ 1–43FX. Biofilms were grown at 37° C in CO_2_ enriched environment. Cells were imaged at 72 h post inoculation. The images represent 3 independent experiments, from each repeat at least 10 fields examined. Scale bar = 2 μm. **(B)** Imaging Flow cytometry analysis of the number of aggregation events, population of the indicated species and **(C)** aggregate size-the mean of measured area of bacterial aggregates population. Cells were grown in liquid TSB medium supplemented with glucose (1% W/V), raffinose (1% W/V), and TSB medium supplemented with glucose (1% W/V) + buffer. Data were collected from overnight culture, and 20,000 cells were counted. Y-axis represents the % of aggregate events or area mean, and graphs represent mean ± SD from 2 independent experiments (*n* = 6). Statistical analysis was performed using Brown-Forsythe and Welch’s ANOVA with Dunnett’s T3 multiple comparisons test. *p* < 0.05 was considered statistically significant.

To better assess the level of changes on the single-cell level we grew the bacteria on solid growth media (TSB), TSB with glucose, and TSB with raffinose in the presence and absence of a buffering system. Colony cells grown without glucose are rod-shaped, divide normally, and are arranged in short chains (Figure 5A). While all the species looked similar in TSB, glucose-induced noticeable alterations in the morphology of LGG and *L. paracasei* cells were consistent with the observed alterations in cell aggregation properties. Noticeable clumps were induced in both species, which also lost their characteristic elongated rod shape. Alteration in cell shape upon glucose treatment could be partially rescued with the application of buffer, supporting a differential response of probiotic bacteria to fermentation and its acidic products. Interestingly, alterations in cell clumping did not perfectly correlate with the response to acid stress as judged by cell growth as LGG had pronounceable growth upon utilization of glucose, while *L. paracasei* failed to increase it carrying capacity with glucose. To further assess whether alterations in cell properties account for the differential colony morphology, we used imaging flow cytometry (Narayana et al., 2020; Maan et al., 2021). While imaging flow cytometry was sporadically used to monitor aggregation (Konieczny et al., 2021), it was never applied to probiotic bacteria.

For each bacterium, we could easily differentiate between single cells and small aggregates vs. larger aggregates (Supplementary Figures S4–S9), and to confirm with high accuracy the % of aggregation evens out of the total measured events. As each bacterium has different aggregation properties, the population of aggregates was determent based on the area value. In LGG, *L. paracasei* and *B. coagulans* we could not fully separate aggregates from bacterial chains. Our results indicated (Figures 5B,C) that LAB strains greatly differ in their basal aggregation properties as judged by the portion of the population capable to aggregate and the average size of the observed aggregates (Figure 5B; Supplementary Figures S4–S9). Overall, the frequency of aggregation properties (Figure 5B) was more in response to the carbohydrate’s composition than the size of the aggregate (Figure 5C) which was an intrinsic property of the probiotic strain. Glucose (but not raffinose) significantly induced aggregation in all Lactobacillaceae strains, except for *L. acidophilus*, in which both glucose and raffinose induced aggregation. For *B. coagulans*, neither glucose nor raffinose affects the formation of aggregates (Figure 5B), indicating that carbohydrate-driven aggregation is a unifying response in Lactobacillaceae that preferentially occurs with glucose. This carbohydrate-driven aggregation was not rescued with buffering, consistent with the partial effect of buffering on cell death within biofilm colonies and the 3D structure of the colonies (Figure 4).

**Figure 6:**
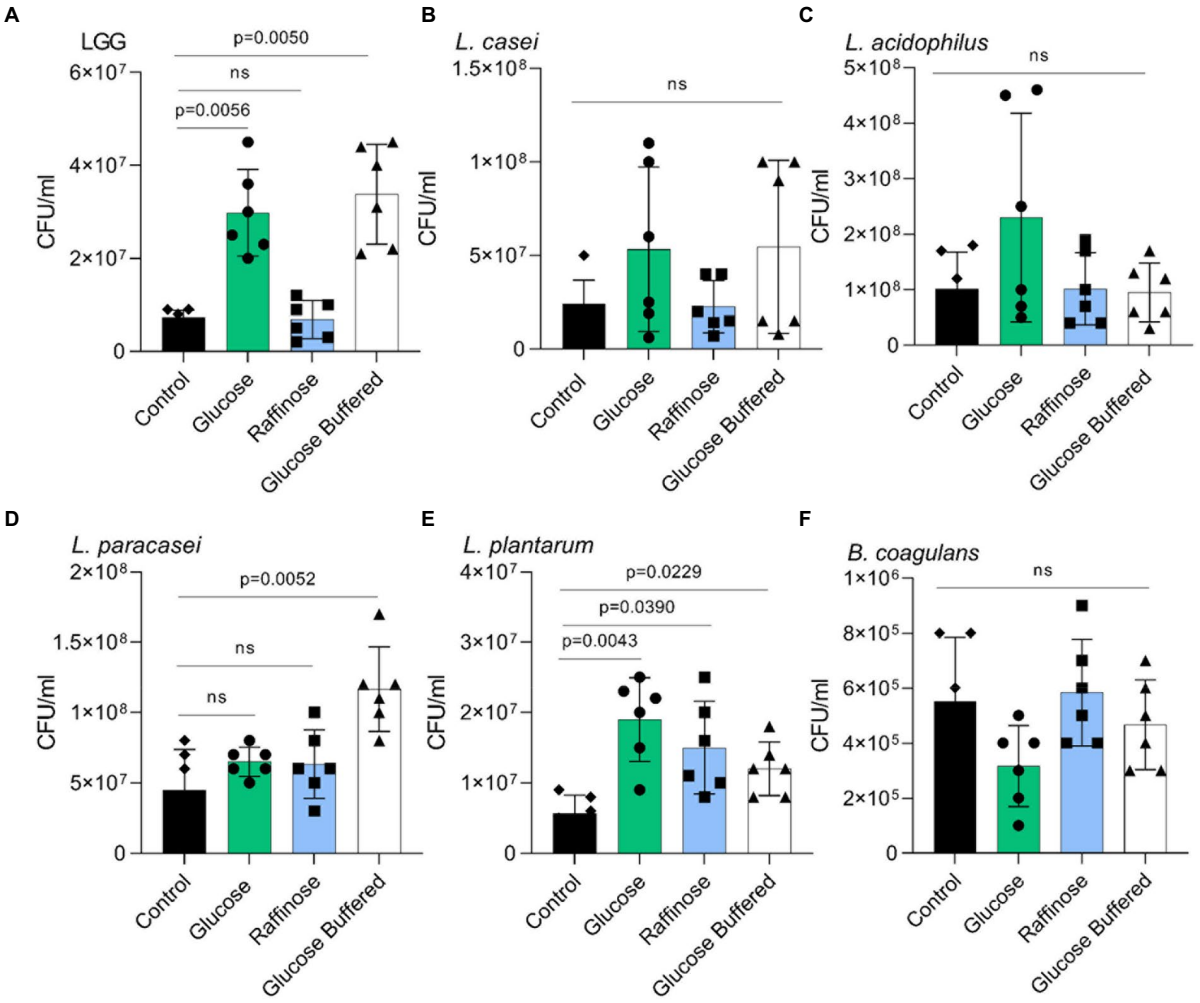
Glucose but not acid stress promotes adhesion to mucin. Adherence of the indicated species to porcine mucin in a microtiter plate. **(A) LGG**. **(B)** *L. casei*. **(C)** *L. acidophilus* **(D)** *L. paracasei*. **(E)** *L. plantarum* **(F)** *B. coagulans* Y-axis represents the number of bacteria adherent to mucin by CFU/ml, graphs represent mean ± SD from 2 independent experiments (n = 6). Statistical analysis was performed using Brown-Forsythe and Welch’s ANOVA with Dunnett’s T3 multiple comparisons test. p < 0.05 was considered statistically significant.

In a host, LAB strains must adhere to the host’s tissue, frequently adhering to mucins. Therefore, we tested whether the overall changes in the adhesive properties of LAB strains and, in particular, a specific response to glucose but not raffinose were observed in LGG. Interestingly, raffinose was an efficient inducer of adhesion for *L. plantarum*. The basal level of adhesion to mucin primarily differed between LAB strains, as did aggregation (Figure 6). The effect of buffering on glucose-dependent adhesion was insignificant, indicating that adhesion is not triggered by the acidification of the medium but is a specific carbohydrate-mediated response.

## Discussion

Probiotic bacteria are considered a means for microbiota modulation, along with nutrition, personal hygiene, and lifestyle (Spor et al., 2011). Probiotics are found to have a beneficial effect on the consumer in the prevention of antibiotic-associated diarrhea and acute infectious diarrhea, treatment of inflammatory bowel disease (Saez-lara et al., 2015), and other gastric disorders (O’Mahony et al., 2005). Lactobacillaceae are widely used probiotic bacteria, represented in most fermented products and supplements. Probiotic performance in the gut is dependent on nutrient composition and availability (Singh et al., 2017). Positive effects on probiotics proliferation and beneficial effects on the host have been associated with prebiotic consumption. For example, it was shown that the addition of raffinose and *L. acidophilus* to the diet of rats decreases their body weight and increases the concentration of *L. acidophilus* in the gut (1-s2.0-S027153179600231X-main). However, the responses of single LAB strains to their acidic fermentation products may vary and contribute to their physiological responses to carbohydrates.

Therefore, we systematically compared five different LAB species for their response to the utilization of glucose and raffinose model sugars. We found that glucose utilization has a general role in shaping the colony morphology under static conditions, as all species exhibit morphological colony changes upon glucose treatment. Similarly, medium acidification was generally similar during static growth (Figure 4B) and comparable for all Lactobacillaceae during planktonic growth (Figure 2). For all tested species, the final product of glucose utilization and fermentation (occurring under microaerophilic conditions) was almost exclusively the organic acid lactate alongside acetate. Analyses of the fermentation capacities of the different species revealed similar fermentation efficiencies. Lactic and acetic acid concentrations and the pH of the growth medium were all similar and stable among the different species (Figures 3,4B). In agreement, the alignment of lactate dehydrogenase proteins revealed that LGG*, L. paracasei,* and *L. casei* have two groups of almost identical protein homologs with more than 90 % identity between the different species (Supplementary Figure S3). This indicates that differential utilization of glucose may not account for the differential carrying capacities of the cultures in the presence of glucose.

Contrary to glucose utilization, organic acid accumulation, and medium acidification, which are comparable between strains, the carrying capacity of the culture is differentially enhanced during the planktonic growth of Lactobacillaceae. While LGG*, L. acidophilus L. casei* and *L. plantarum* doubled the carrying capacity following exposure to glucose exposure, *L. paracasei* had no noticeable growth induction with glucose. Interestingly, the capacity of LAB strains to utilize glucose, raffinose, mannose, and xylose for planktonic growth differed significantly (Figures 1,2, Supplementary Figure S1). A detailed analysis of the microbial growth reflected that carbon utilization is unintuitively inert to the growth rate, and does not affect the lag phase (Figure 2). Rather, it allows the bacterial community to reach a significantly higher carrying capacity that may account for changes in their proportions in the GI.

Acid stress in Lactobacillaceae is self-imposed stress, and, thus, LAB is relatively acid-tolerant, and employs several mechanisms to regulate the homeostasis of the pH level (De Angelis and Gobbetti, 2004). The mechanisms generally include the removal of protons or alkalization of the environment *via* ammonia production through arginine deiminase (ADI; Costa, 2002). In addition, glutamic acid decarboxylase (GAD) catalyzes the decarboxylation of glutamate into gamma-aminobutyric acid (GABA), which results in the alkalization of the cytoplasmic pH due to the removal of protons (Gobbetti et al., 2010). Lastly, the urease system allows the hydrolysis of urea, which enhances the survival of LAB under acid stress conditions by the production of NH_3_. The urease operon was found to be positively regulated under low pH levels in the LAB *Streptococcus salivarius* (Huang et al., 2014). The F-ATPase system is another mechanism that protects LAB from acid damage. Overall, probiotic acid-tolerant species are of great value as additional encapsulation to ensure survival to transit through the acidic stomach. As the survival of LAB during acid stress is quite heterogeneous the expression of these systems during self-imposed acid stress and, in the stomach, needs to be properly evaluated while selecting formulating probiotics. Alternatively, food carriers with high buffering potential to aid gastric transit and ensure that viable cells reach the small intestine may contribute to assure a beneficial effect on the host. For example, our results indicate that *L. paracasei* in liquid (Figure 1) and *L. acidophilus* (Figure 4) grown on a solid medium exhibit poor acid tolerance during self-imposed acid stress. One immediate application of our findings for *L. paracasei* in solution (Figure 1) and *L. acidophilus* (Figure 4C) is that the probiotic performance of these strains in the gut is dependent on the availability of a suitable buffering system.

In the gut, acid-sensitive species are protected from the acidic pH of the stomach (2–4) because of the buffering properties of food, which depend on the type of food and its volume (Simonian et al., 2005; Papadimitriou et al., 2015). In parallel, self-imposed acid stress from the accumulation of organic acids following the utilization of carbohydrates may affect the performance of the ingested bacterial species. Interestingly, buffering of the colonies restored the glucose-free morphology of all strains, indicating that acid stress is involved in biofilm formation throughout the Firmicutes phylum directly or indirectly. The addition of a buffering system to the growth media of bacteria grown in the presence of glucose increased the survival of most species significantly. However, while cell death was decreased with buffering, it was still significantly higher with glucose compared with non-glucose conditions (Figure 4C). Thus, the glucose-induced change in colony morphology may be reflective of a complex adaptation and biofilm regulation, rather than a simple reflection of cell density.

Our results, observed in all LAB strains are consistent with recent findings that the stress protein Hsp plays an important role in shaping colony morphology under acidic pH in a single specie: *L. plantarum* (Rajasekharan and Shemesh, 2022). While glucose utilization capacities and medium acidification under our conditions were similar, the enhancement of carrying capacity (Figure 2), carbohydrate-mediated cell death within the colony (Figure 4C), and the adaptation to glucose utilization, are reflected by enhanced aggregation properties (Figure 5) varied dramatically between the tested species. The observed alterations in cell shape and aggregation properties may indicate an independent adaption to fermentable carbohydrates (for example, a change in surface proteome) and not a direct reflection of acid stress as they poorly responded to buffering.

Changes in the surface proteome (Savijoki et al., 2019), quorum sensing (Di Cagno et al., 2011), and the activation of differential target genes of the stressosome (Papadimitriou et al., 2016) may differ between LAB strains. These results are consistent with pH independent changes in the aggregation and adhesion properties of the LAB strains tested with glucose. Raffinose was an efficient inducer of aggregation of *L. acidophilus* (Figure 5B) and adhesion of *L. plantarum* (Figure 6). These exceptions indicate that specific response to carbohydrates as mediators of cellular adhesive properties is not limited to glucose or highly fermentable sugars and that additional layers of carbohydrate-responsive adhesins/cell envelope components evolved in the microbiome. While the exact regulations remain to be determined, our results indicate that the overall physiological response to carbohydrate metabolism and self-imposed acid stress is not a mere reflection of microbial growth.

## Materials and methods

### Strains, media, and imaging

*Lactobacillus acidophilus* ATCC 4356, *Lacticaseibacillus casei* ATCC 393, *Lacticaseibacillus casei subsp. paracasei* ATCC BAA-52, *Lactiplantibacillus plantarum* ATCC 8014, and *Lacticaseibacillus rhamnosus GG* ATCC 53103 probiotic strains were used in the study. *Bacillus coagulans* ATCC 10545 was used as a control. A single colony of Lactobacillaceae was isolated on a solid deMan, Rogosa, Sharpe Agar (MRS) plate, inoculated into 5 ml MRS broth (Difco, Le Pont de Claix, France), and grown at 37°C, without shaking overnight. A single colony of *Bacillus coagulans* isolated on a solid LB agar plate was inoculated into 5 ml LB broth (Difco) and grown at 37°C, with shaking overnight. For biofilm colonies, these cultures were inoculated into a solid medium (1.5% agar) containing 50% Tryptic soy broth (TSB), TSB supplemented with (1% w/v) D-(+) - glucose (1% w/v) D-(+)- raffinose or (1% w/v) D-(+)- mannose or (1% w/v) D-(+)-Xylose or with (1% w/v) D-(+) - glucose buffered with MOPS (3-(N-morpholino)propane-sulfonic acid) and potassium phosphate buffer. The bacteria were incubated in a BD GasPak EZ - Incubation Container with BD GasPak EZ CO_2_ Container System Sachets (260679; Becton, Sparks, MD, United States), for 72 h at 37°C. The colony images were taken using a Stereo Discovery V20″ microscope (Tochigi, Japan) with objectives Plan Apo S × 1.0 FWD 60 mm (Zeiss, Goettingen, Germany) attached to a high-resolution microscopy Axiocam camera. Data were created and processed using Axiovision suite software (Zeiss). For planktonic growth, the bacterial cultures were inoculated into a liquid medium of 50% TSB with different sugars as described above, and incubated for 24 h, at 37°C.

### Growth measurement and analysis

Cultured cells grown overnight were diluted 1:100 in 200 μl liquid medium contains 50% TSB (BD), TSB supplemented with (1% w/v) D-(+)- glucose, (1% w/v) D-(+)- raffinose or (1% w/v) D-(+)- mannose or (1% w/v) D-(+)-Xylose in a 96-well microplate (Thermo Scientific, Roskilde, Denmark). Cells were grown with agitation at 37°C for 18 h in a microplate reader (Tecan, Männedorf, Switzerland), and the optical density at 600 nm (OD_600_) was measured every 30 min. Maximum OD, growth rate, and lag time calculation were performed with GrowthRates 3.0 software.

### Fluorescence microscopy

A bacterial colony grown as described above was suspended in 200 μl 1x Phosphate-Buffered Saline (PBS), and dispersed by pipetting. Samples were centrifuged briefly, pelleted, and re-suspended in 5 μl of 1x PBS supplemented with the membrane stain FM1-43 (Molecular Probes, Eugene, OR, United States) at 1 μg/ml. These cells were placed on a microscope slide and covered with a poly-L-Lysine (Sigma) treated coverslip. The cells were observed by Axio microscope (Zeiss, Germany). Images were analyzed by Zen-10 software (Zeiss).

### pH measurements

After inoculation in different liquid mediums and 24 h incubation (without shaking) at 37°C, the cells were separated from the medium by centrifugation (4,000 × *g*, 20 min) followed by filtration through a 0.22 μm. The conditioned media acidity was measured by the pH meter (Mettler Toledo). pH measurements of the solid media were done using pH-indicator strips (MQuant®, Merck KGaA, Darmstadt, Germany).

### Determination of organic acids

The supernatant of the bacteria grown on a defined medium-MSgg with 1% glucose for 24 h was filtered through 0.22 μm filter membranes for HPLC analysis. The contents of organic acids in each liquid sample were determined using an Infinity 1260 (Agilant Technologies, Santa Clara, CA, United States) HPLC system with C 18 column (Syncronis™ C18 Columns, 4.6 × 250 mm, 5 μm). Mobile phase A was acetonitrile and mobile phase B was 5-mM KH_2_PO_4_ pH 2.4. The flow rate was kept constant at 1 ml/min, with ultraviolet detection performed at 210 nm. The injection volume was 20 μl and the column temperature was maintained at 30°C. Identification and quantification of organic acids were accomplished by comparing the retention times and areas with those of pure standards.

Notably, although quantification of organic acids in TSB was not feasible due to the high background, the comparable acidity strongly indicates similar fermentation capacities of the strains tested in rich growth media.

### Flow cytometry analysis

Starter cultures were spotted on TSB with glucose or TSB with glucose buffered with MOPS and potassium phosphate buffer. The plates were then incubated as mentioned in the first section. Colonies were harvested after 72 h and separated with mild sonication. Samples were diluted in PBS and measured using an LSR-II new cytometer (Becton Dickinson, San Jose, CA, United States). PI (Propidium Iodide, 20 Mm, Invitrogen™) fluorescence was measured using laser excitation of 488 nm, coupled with 600 LP and 610/20 sequential filters. A total of 100,000 cells were counted for each sample and flow cytometry analyses were performed using BD FACSDiva software.

### Imaging flow cytometry for aggregation

Strains were grown in different liquid mediums for overnight incubation (without shaking) at 37°C. Data were acquired by ImageStreamX Mark II (AMNIS, Austin, TX) using a 60× lens (NA = 0.9). The laser used was 785 nm (5 mW) for side scatter measurement. During acquisition, bacterial cells were gated according to their area (in square microns) and side scatter, which excluded the calibration beads (that run in the instrument along with the sample). For each sample, 20,000 events were collected. Data were analyzed using IDEAS 6.2 (AMNIS). Focused events were selected by the Gradient RMS, a measurement of image contrast. Singlets and small aggregates events vs. larger microbial aggregates and chain events were selected according to their area (in square microns) and aspect ratio (width divided by the length of the best fit ellipse) of the bright filed image. Aggregate event populations were determined for each bacterium, from an Area value of 55 for LGG, 25 for *L. casei*, 27 for *L. acidophilus*, 68 for *L. paracasei*, 24 for *L. plantarum* and 35 for *B. coagulans* (Supplementary Figures S4–S9). The size of the population of the aggregate event was quantified using the Area feature (the number of microns squared in a mask, in μm^2^) of the brightfield image.

### Mucin adhesion assay

Strains were assayed for adhesion to mucin in 96-well microtiter plates under sterile conditions. Plates were coated with 100 μl of 10 mg/ml porcine Mucin Type || (Sigma-Aldrich) in sterile Dulbecco’s phosphate-buffered saline (PBS) at 4°C overnight. Wells were washed twice with sterile PBS to remove unbound mucin. Strains were grown in different liquid mediums for overnight incubation (without shaking) at 37°C. The cells grown overnight were harvested by centrifugation (10,000 × *g* for 2 min at 4°C) and the bacterial cells were resuspended in sterile PBS and adjusted to the optical density (OD_600_) of 0.5. 100 μl of each strain was added to respective wells and allowed to adhere for 2 h at 37°C. Un-adhered bacterial cells were then withdrawn, and wells were washed 3 times with 100 μl sterile PBS each. Adhered cells were released by treatment with 100 μl 0.1% (v/v) Triton X-100 in sterile PBS for 30 min at 37°C. The released bacterial cells were plated after appropriate dilution on MRS agar, and enumeration was carried out following 48-h incubation at 37°C.

### Protein alignment, phylogenetic tree

A phylogenetic tree was built based on 16S rRNA bacterial gene sequences and multiple alignment sequences of LDH proteins was performed using Clustal Omega.^1^

### Statistical analysis

All experiments were performed at least three separate and independent times in triplicate unless stated otherwise. Statistical analyses were performed with GraphPad Prism 9.0 (GraphPad 234 Software, Inc., San Diego, CA). Relevant statistical tests are mentioned in the indicated legends of the figures.

## Data availability statement

The original contributions presented in the study are included in the article/Supplementary material, further inquiries can be directed to the corresponding author.

## Author contributions

IKG, OK, MM, RS, and RO designed the experiments. RS and RO performed the experiments. IKG, MM, OK, HM, OG, RS, and RO contributed reagents. All authors analyzed the data. IKG wrote the paper. All authors contributed to the article and approved the submitted version.

## Funding

This work was funded by ImoH grant 3-15656 and ISF 119/16 to IKG.

## Conflict of interest

The authors declare that the research was conducted in the absence of any commercial or financial relationships that could be construed as a potential conflict of interest.

## Publisher’s note

All claims expressed in this article are solely those of the authors and do not necessarily represent those of their affiliated organizations, or those of the publisher, the editors and the reviewers. Any product that may be evaluated in this article, or claim that may be made by its manufacturer, is not guaranteed or endorsed by the publisher.

## Footnotes

^1^ https://www.ebi.ac.uk/Tools/msa/clustalo/

## References

[ref2] Azad M. A. K. Sarker M. Li T. Yin J. (2018). Probiotic species in the modulation of gut microbiota: an overview. Biomed. Res. Int. 2018, 1–8. doi: 10.1155/2018/9478630, PMID: 29854813 PMC5964481

[ref3] Bintsis T. (2018). Lactic acid bacteria as starter cultures: an update in their metabolism and genetics. AIMS Microbiol. 4, 665–684. doi: 10.3934/MICROBIOL.2018.4.665, PMID: 31294241 PMC6613329

[ref4] Cao J. Yu Z. Liu W. Zhao J. Zhang H. Zhai Q.. (2020). Probiotic characteristics of Bacillus coagulans and associated implications for human health and diseases. J. Funct. Foods 64:103643. doi: 10.1016/J.JFF.2019.103643

[ref5] Celebioglu H. U. Ejby M. Majumder A. Købler C. Goh Y. J. Thorsen K.. (2016). Differential proteome and cellular adhesion analyses of the probiotic bacterium Lactobacillus acidophilus NCFM grown on raffinose: an emerging prebiotic. Proteomics 16, 1361–1375. doi: 10.1002/pmic.201500212, PMID: 26959526

[ref6] Costa C. M. (2002). Formulário para parecer. Projeto de Pesquisa 68, 6193–6201. doi: 10.1128/AEM.68.12.6193

[ref7] De Angelis M. Gobbetti M. (2004). Environmental stress responses in lactobacillus: a review. Proteomics 4, 106–122. doi: 10.1002/pmic.200300497, PMID: 14730676

[ref8] Di Cagno R. De Angelis M. Calasso M. Gobbetti M. (2011). Proteomics of the bacterial cross-talk by quorum sensing. J. Proteome 74, 19–34. doi: 10.1016/J.JPROT.2010.09.003, PMID: 20940064

[ref9] Didari T. Solki S. Mozaffari S. Nikfar S. Abdollahi M. (2014). A systematic review of the safety of probiotics. Expert Opin. Drug Saf. 13, 227–239. doi: 10.1517/14740338.2014.872627 24405164

[ref10] Ferain T. Garmyn D. Bernard N. Hols P. Jean Delcour A. (1994). Lactobacillus plantarum ldhL gene: overexpression and deletion. J. Bacteriol. 176, 596–601. 8300514 10.1128/jb.176.3.596-601.1994 PMC205095

[ref11] Fontana L. Bermudez-Brito M. Plaza-Diaz J. Muñoz-Quezada S. Gil A. (2004). Sources, isolation, characterisation and evaluation of probiotics. Br. J. Nutr. 109:S35–S50. doi: 10.1017/S0007114512004011 23360880

[ref12] Garro M. S. De Valdez G. F. Oliver G. De Giori G. S. (1998). Growth characteristics and fermentation products of Streptococcus salivarius subsp. thermophilus, Lactobacillus casei and L. fermentum in soymilk. Eur. Food Res. Technol. 206, 72–75. doi: 10.1007/s002170050217

[ref13] Gobbetti M. Di Cagno R. de Angelis M. (2010). Functional microorganisms for functional food quality. Crit. Rev. Food Sci. Nutr. 50, 716–727. doi: 10.1080/10408398.2010.499770 20830633

[ref14] Hall B. G. Acar H. Nandipati A. Barlow M. (2014). Growth rates made easy. Mol. Biol. Evol. 31, 232–238. doi: 10.1093/MOLBEV/MST187 24170494

[ref15] Hedberg M. Hasslöf P. Sjöström I. Twetman S. Stecksén-Blicks C. (2008). Sugar fermentation in probiotic bacteria: an in vitro study. Oral Microbiol. Immunol. 23, 482–485. doi: 10.1111/j.1399-302X.2008.00457.x, PMID: 18954354

[ref16] Hofvendahl K. Hahn-Hägerdal B. (2000). Factors affecting the fermentative lactic acid production from renewable resources. Enzyme Microb. Technol. 26, 87–107. 10689064 10.1016/s0141-0229(99)00155-6

[ref17] Hols P. (2004). Major role of NAD-dependent lactate dehydrogenases in aerobic lactate utilization in Lactobacillus plantarum during early stationary phase. Microbiology 186, 6661–6666. doi: 10.1128/JB.186.19.6661, PMID: 15375150 PMC516598

[ref18] Huang S. C. Burne R. A. Chen Y. Y. M. (2014). The pH-dependent expression of the urease operon in Streptococcus salivarius is mediated by CodY. Appl. Environ. Microbiol. 80, 5386–5393. doi: 10.1128/AEM.00755-14, PMID: 24951785 PMC4136106

[ref19] Huang Y. Xin W. Xiong J. Yao M. Zhang B. Zhao J. (2022). The intestinal microbiota and metabolites in the gut-kidney-heart axis of chronic kidney disease. Front. Pharmacol. 13:734. doi: 10.3389/FPHAR.2022.837500/BIBTEX PMC8971625 35370631

[ref20] Karapetsas A. Vavoulidis E. Galanis A. Sandaltzopoulos R. Kourkoutas Y. (2010). Rapid detection and identification of probiotic Lactobacillus casei ATCC 393 by multiplex PCR. Microb. Physiol. 18, 156–161. doi: 10.1159/000308518 20389120

[ref21] Konieczny M. Rhein P. Czaczyk K. Białas W. Juzwa W. (2021). Imaging flow cytometry to study biofilm-associated microbial aggregates. Molecules 26, 1–12. doi: 10.3390/molecules26237096, PMID: 34885675 PMC8659131

[ref22] Maan H. Gilhar O. Porat Z. Kolodkin-Gal I. (2021). Bacillus subtilis colonization of Arabidopsis thaliana roots induces multiple biosynthetic clusters for antibiotic production. Front. Cell. Infect. Microbiol. 11, 1–10. doi: 10.3389/fcimb.2021.722778, PMID: 34557426 PMC8454505

[ref23] Narayana S. K. Mallick S. Siegumfeldt H. van den Berg F. (2020). Bacterial flow cytometry and imaging as potential process monitoring tools for industrial biotechnology. Fermentation 6:10. doi: 10.3390/fermentation6010010

[ref24] O’Mahony L. Mccarthy J. Kelly P. Hurley G. Luo F. Chen K.. (2005). Lactobacillus and Bifidobacterium in irritable bowel syndrome: symptom responses and relationship to cytokine profiles. Gastroenterology 128, 541–551. doi: 10.1053/j.gastro.2004.11.050, PMID: 15765388

[ref25] Palacio M. I. Etcheverría A. Manrique G. Palacio M. I. Etcheverría A. I. Manrique G. D. (2014). Fermentation by Lactobacillus paracasei of galactooligosaccharides and low-molecular-weight carbohydrates extracted from squash (Curcubita maxima) and lupin (Lupinus albus) seeds. J. Microbiol. Biotechnol. Food Sci. 3, 329–332.

[ref26] Papadimitriou K. Alegría Á. Bron P. A. de Angelis M. Gobbetti M. Kleerebezem M.. (2016). Stress physiology of lactic acid bacteria. Microbiol. Mol. Biol. Rev. 80, 837–890. doi: 10.1128/MMBR.00076-15/ASSET/AF4F2855-634D-46C6-A58C-BAA8988DF6CC/ASSETS/GRAPHIC/ZMR0031624320005.JPEG, PMID: 27466284 PMC4981675

[ref27] Papadimitriou K. Zoumpopoulou G. Foligné B. Alexandraki V. Kazou M. Pot B.. (2015). Discovering probiotic microorganisms: in vitro, in vivo, genetic and omics approaches. Front. Microbiol. 6:58. doi: 10.3389/fmicb.2015.00058, PMID: 25741323 PMC4330916

[ref28] Paucean A. Vodnar D. C. Socaci S. A. Socaciu C. (2013). Carbohydrate metabolic conversions to lactic acid and volatile derivatives, as influenced by Lactobacillus plantarum ATCC 8014 and Lactobacillus casei ATCC 393 efficiency during in vitro and sourdough fermentation. Eur. Food Res. Technol. 237, 679–689. doi: 10.1007/s00217-013-2042-6

[ref29] Povolotsky T. L. Keren-Paz A. Kolodkin-Gal I. (2021). Metabolic microenvironments drive microbial differentiation and antibiotic resistance. Trends Genet. 37, 4–8. doi: 10.1016/J.TIG.2020.10.007, PMID: 33203570

[ref30] Qin J. Li R. Raes J. Arumugam M. Burgdorf K. S. Manichanh C.. (2010). A human gut microbial gene catalogue established by metagenomic sequencing. Nature 464, 59–65. doi: 10.1038/nature08821, PMID: 20203603 PMC3779803

[ref31] Rajasekharan S. K. Shemesh M. (2022). Spatiotemporal bio-shielding of bacteria through consolidated geometrical structuring. NPJ Biofilms Microbiomes 8:37. doi: 10.1038/s41522-022-00302-2, PMID: 35534500 PMC9085766

[ref32] Rocchetti M. T. Russo P. Capozzi V. Drider D. Spano G. Fiocco D. (2021). Bioprospecting antimicrobials from Lactiplantibacillus plantarum: key factors underlying its probiotic action. Int. J. Mol. Sci. 22:12076. doi: 10.3390/IJMS222112076, PMID: 34769500 PMC8585029

[ref33] Saez-lara M. J. Gomez-llorente C. Plaza-diaz J. Gil A. (2015). The role of probiotic lactic acid bacteria and Bifidobacteria in the prevention and treatment of inflammatory bowel disease and other related diseases: a systematic review of randomized human clinical trials. Biomed. Res. Int. 2015:505878. doi: 10.1155/2015/505878, PMID: 25793197 PMC4352483

[ref34] Salas-Jara M. J. Ilabaca A. Vega M. García A. (2016). Biofilm forming lactobacillus: new challenges for the development of probiotics. Microorganisms 4:35. doi: 10.3390/microorganisms4030035, PMID: 27681929 PMC5039595

[ref35] Savijoki K. Nyman T. A. Kainulainen V. Miettinen I. Siljamäki P. Fallarero A.. (2019). Growth mode and carbon source impact the surfaceome dynamics of lactobacillus rhamnosus GG. Front. Microbiol. 10:1272. doi: 10.3389/FMICB.2019.01272/BIBTEX, PMID: 31231350 PMC6560171

[ref36] Segers M. E. Lebeer S. (2014). Towards a better understanding of Lactobacillus rhamnosus GG - host interactions. Microb. Cell Factories 13:S7. doi: 10.1186/1475-2859-13-S1-S7, PMID: 25186587 PMC4155824

[ref37] Simonian H. P. Vo L. Doma S. Fisher R. S. Parkman H. P. (2005). Regional postprandial differences in pH within the stomach and gastroesophageal junction. Dig. Dis. Sci. 50, 2276–2285. doi: 10.1007/s10620-005-3048-0, PMID: 16416175

[ref38] Singh R. K. Chang H. W. Yan D. Lee K. M. Ucmak D. Wong K.. (2017). Influence of diet on the gut microbiome and implications for human health. J. Transl. Med. 15:17. doi: 10.1186/s12967-017-1175-y, PMID: 28388917 PMC5385025

[ref39] Spor A. Koren O. Ley R. (2011). Unravelling the effects of the environment and host genotype on the gut microbiome. Nat. Rev. Microbiol. 9, 279–290. doi: 10.1038/nrmicro2540, PMID: 21407244

[ref40] Turroni F. Ventura M. Buttó L. F. Duranti S. O’Toole P. W. Motherway M. O. C.. (2014). Molecular dialogue between the human gut microbiota and the host: a Lactobacillus and Bifidobacterium perspective. Cell. Mol. Life Sci. 71, 183–203. doi: 10.1007/s00018-013-1318-0, PMID: 23516017 PMC11113728

[ref41] Vijaya Kumar B. Vijayendra S. V. N. Reddy O. V. S. (2015). Trends in dairy and non-dairy probiotic products: a review. J. Food Sci. Technol. 52, 6112–6124. doi: 10.1007/s13197-015-1795-2, PMID: 26396359 PMC4573104

[ref42] Wang Y. Wu J. Lv M. Shao Z. Hungwe M. Wang J.. (2021). Metabolism characteristics of lactic acid bacteria and the expanding applications in food industry. Front. Bioeng. Biotechnol. 9:378. doi: 10.3389/FBIOE.2021.612285/BIBTEX PMC8149962 34055755

[ref43] Yeo S. K. Liong M. T. (2010). Effect of prebiotics on viability and growth characteristics of probiotics in soymilk. J. Sci. Food Agric. 90, 267–275. doi: 10.1002/jsfa.3808, PMID: 20355041

[ref44] Zartl B. Silberbauer K. Loeppert R. Viernstein H. Praznik W. Mueller M. (2018). Fermentation of non-digestible raffinose family oligosaccharides and galactomannans by probiotics. Food Funct. 9, 1638–1646. doi: 10.1039/c7fo01887h, PMID: 29465736

[ref45] Zielińska D. Kolozyn-Krajewska D. Laranjo M. (2018). Food-origin lactic acid bacteria may exhibit probiotic properties: review. Biomed. Res. Int. 2018, 1–15. doi: 10.1155/2018/5063185, PMID: 30402482 PMC6191956

