# Supplementary material for: Context-dependent differences in the functional responses of Lactobacillaceae strains to fermentable sugars
Source: Front Microbiol. 2022 Oct 24;13:949932. doi: 10.3389/fmicb.2022.949932 (PMC9637956; doi:10.3389/fmicb.2022.949932)
Supplement: Supplementary file 1 [file Data_Sheet_1.PDF]

Context-dependent differences in the functional responses of Lactobacillaceae strains to fermentable sugars

Supporting Information

Supporting Figures (S1-S9)

Supporting Table (S1)

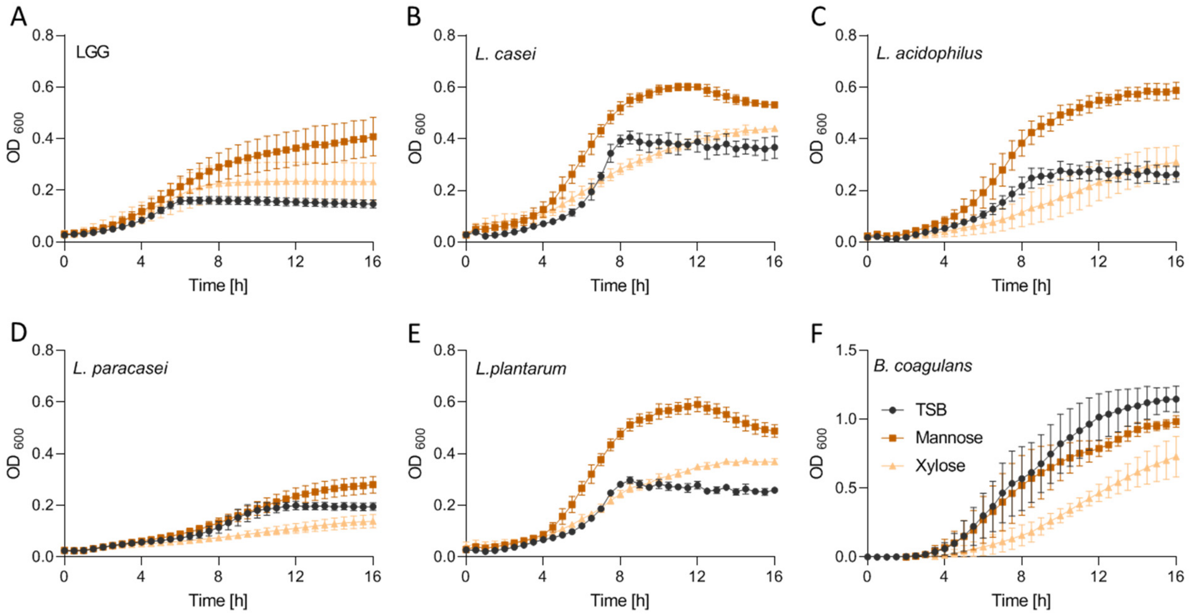

Figure S1. The effect of fermentable sugars on planktonic growth.

Planktonic growth of A) LGG; B) *L. casei*; C) *L. acidophilus*; D) *L. paracasei*; E) *L. plantarum* and F) *B. coagulans* in TSB medium (control) and TSB medium supplemented with mannose (1% W/V) and xylose (1% W/V). Graphs represent mean ± SD from three independent experiments (n=3).

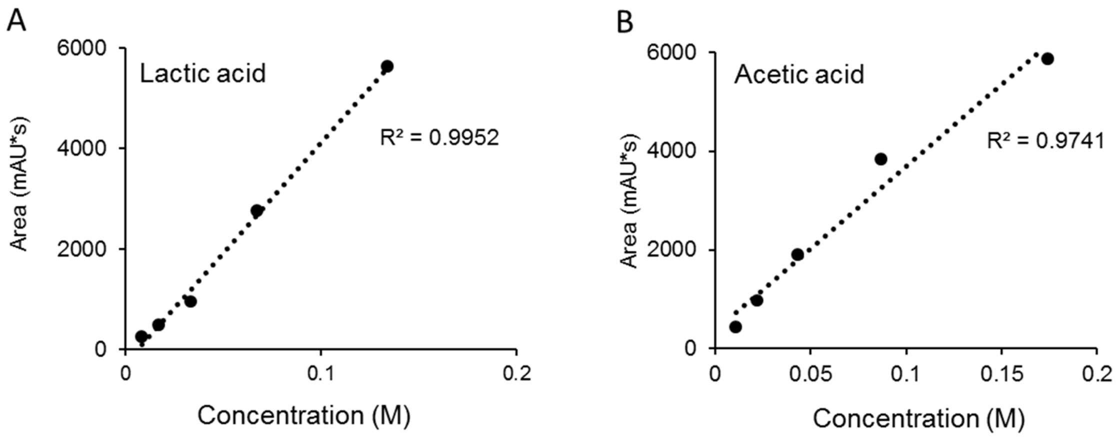

Figure S2. Calibration curves for A) Lactic acid and B) Acetic acid were made using serial dilutions of the acid standard.

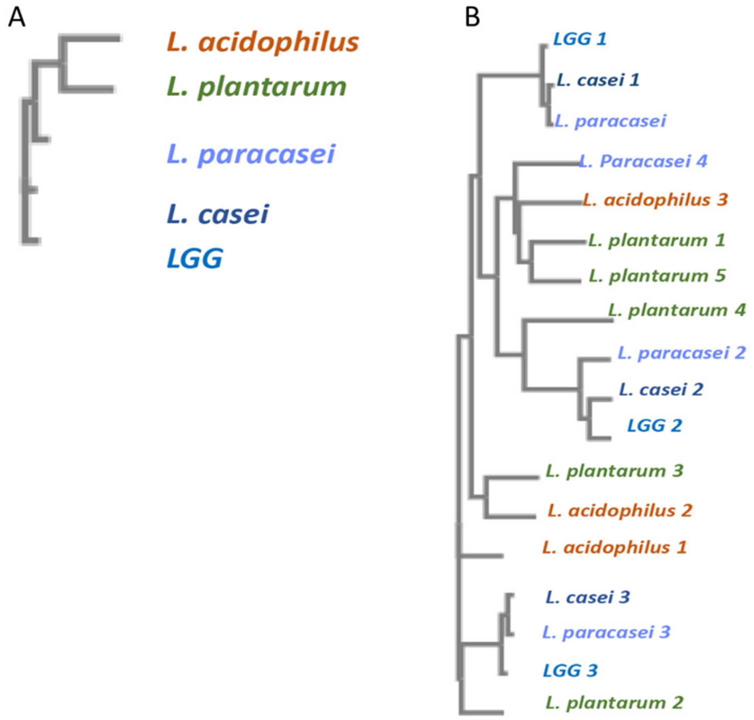

Figure S3. Lactobacillaceae evolutionary distance. **A)**16S or **B)** L- LDH protein based phylogenetic trees were generated using Clustal Omega(1).

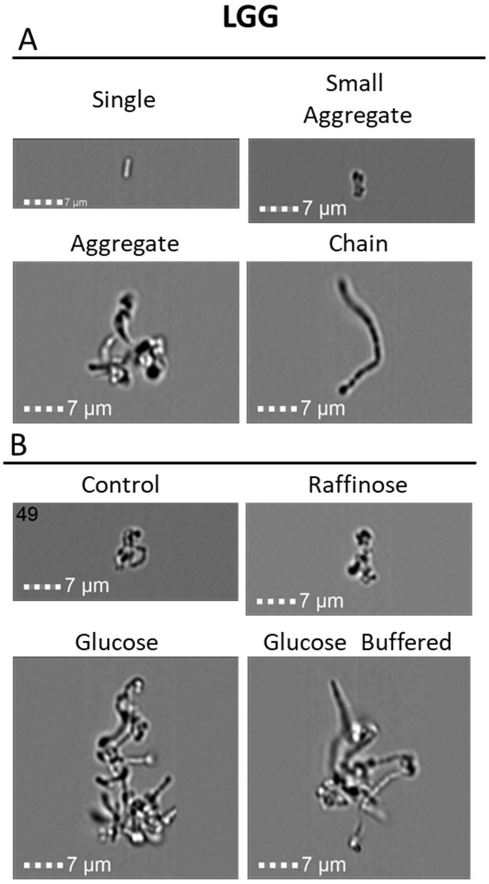

Figure S4. Imaging flow cytometry of LGG cultures. A) LGG representative bright-field images differentiate between single cells and small aggregates (Number of cells <= 3) vs. larger aggregates and chains. **B)** Representative bright-field images of aggregation events population in TSB medium (control), TSB medium supplemented with glucose and raffinose (1% W/V), and TSB medium supplemented with glucose (1% W/V) + buffer.

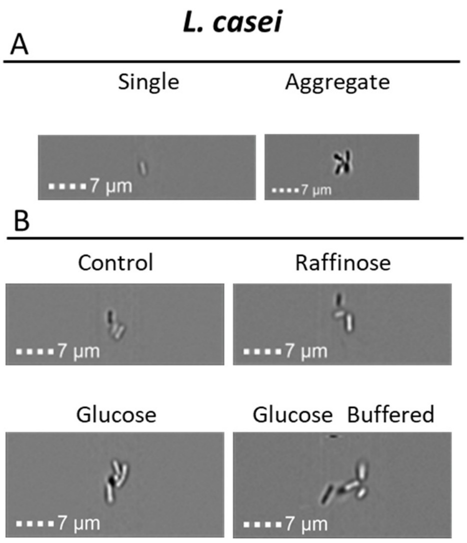

Figure S5. Imaging flow cytometry of *L. casei* cultures. A) L. casei representative bright-field images differentiate between single cells and small aggregates (Number of cells <= 3) vs. larger aggregates including chains. **B)** Representative bright-field images of aggregation events population in TSB medium (control), TSB medium supplemented with glucose and raffinose (1% W/V), and TSB medium supplemented with glucose (1% W/V) + buffer.

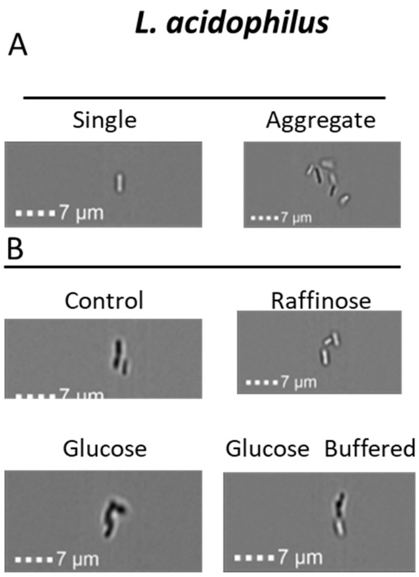

Figure S6. Imaging flow cytometry of *L. acidophilus* cultures. A) *L. acidophilus* representative bright-field images related to differentiate between single cells and small aggregates (Number of cells <= 3) vs. larger aggregates and chains. **B)** Representative bright-field images of aggregation events population in TSB medium (control), TSB medium supplemented with glucose and raffinose (1% W/V), and TSB medium supplemented with glucose (1% W/V) + buffer

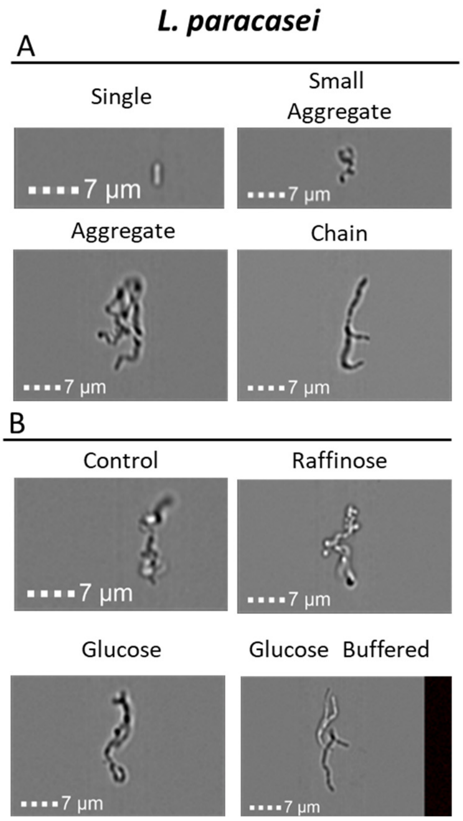

Figure S7. Imaging flow cytometry of *L. paracasei* cultures. A) *L. paracasei* representative bright-field images related to differentiate between single cells and small aggregates (Number of cells <= 3) vs. larger aggregates and chains. **B)** Representative bright-field images of aggregation events population in TSB medium (control), TSB medium supplemented with glucose and raffinose (1% W/V), and TSB medium supplemented with glucose (1% W/V) + buffer

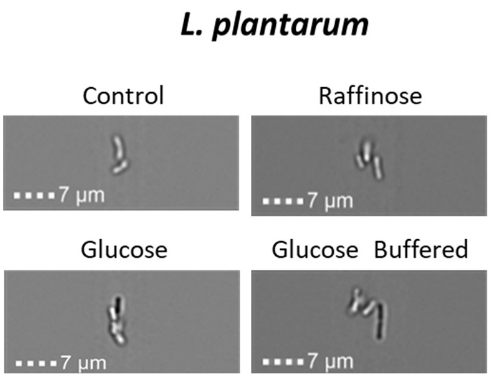

Figure S8. Imaging flow cytometry of *L. plantarum* cultures. Representative bright-field images of aggregation events population in TSB medium (control), TSB medium supplemented with glucose and raffinose (1% W/V), and TSB medium supplemented with glucose (1% W/V) + buffer

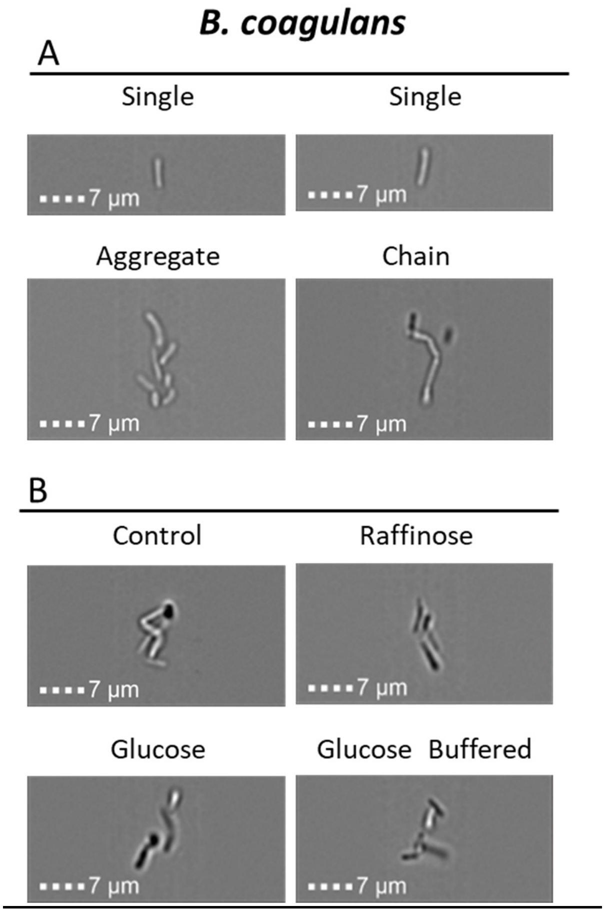

Figure S9. Imaging flow cytometry of *B. coagulans* cultures. A) *B. coagulans* representative bright-field images related to differentiate between single cells and small aggregates (Number of cells <= 3) vs. larger aggregates and chains. **B)** Representative bright-field images of aggregation events population in TSB medium (control), TSB medium supplemented with glucose and raffinose (1% W/V), and TSB medium supplemented with glucose (1% W/V) + buffer

	TSB	TSB	TSB	Glucose	Glucose	Glucose	Raffinose	Raffinose	Raffinose
LGG	6	6	6	4	4	4	6	6	6
L. casei	6	6	6	4	4	4	5	5	5
L. acidophilus	6	6	6	4	4	4	5	5	5
L. paracasei	6	6	6	4	4	4	5	5	5
L. plantarum	6	6	6	4	4	4	5	5	5
B.coagulans	6	6	6	4.5	4.5	5	5.5	5.5	5.5

Table S1. Detailed pH measurements indicated in figure 4B.

Reference

1. Sievers F, Higgins DG. 2014. Clustal Omega. Curr Protoc Bioinforma 48:3.13.1-3.13.16.
